# Gibberellin Metabolism in Flowering Plants: An Update and Perspectives

**DOI:** 10.3389/fpls.2020.00532

**Published:** 2020-05-19

**Authors:** Juan He, Peiyong Xin, Xueting Ma, Jinfang Chu, Guodong Wang

**Affiliations:** ^1^State Key Laboratory of Plant Genomics and National Center for Plant Gene Research, Institute of Genetics and Developmental Biology, The Innovative Academy of Seed Design, Chinese Academy of Sciences, Beijing, China; ^2^College of Advanced Agricultural Sciences, University of Chinese Academy of Sciences, Beijing, China

**Keywords:** gibberellins, plant development, metabolism, hydroxylation, oxidation

## Abstract

In plants, gibberellins (GAs) play important roles in regulating growth and development. Early studies revealed the large chemodiversity of gibberellins in plants, but only GA_1_, GA_3_, GA_4_, and GA_7_ show biological activity that controls plant development. However, the elucidation of the GA metabolic network at the molecular level has lagged far behind the chemical discovery of GAs. Recent advances in downstream GA biosynthesis (after GA_12_ formation) suggest that species-specific gibberellin modifications were acquired during flowering plant evolution. Here, we summarize the current knowledge of GA metabolism in flowering plants and the physiological functions of GA deactivation, with a focus on GA 13 hydroxylation. The potential applications of GA synthetic biology for plant development are also discussed.

## Introduction

**FIGURE 1:**
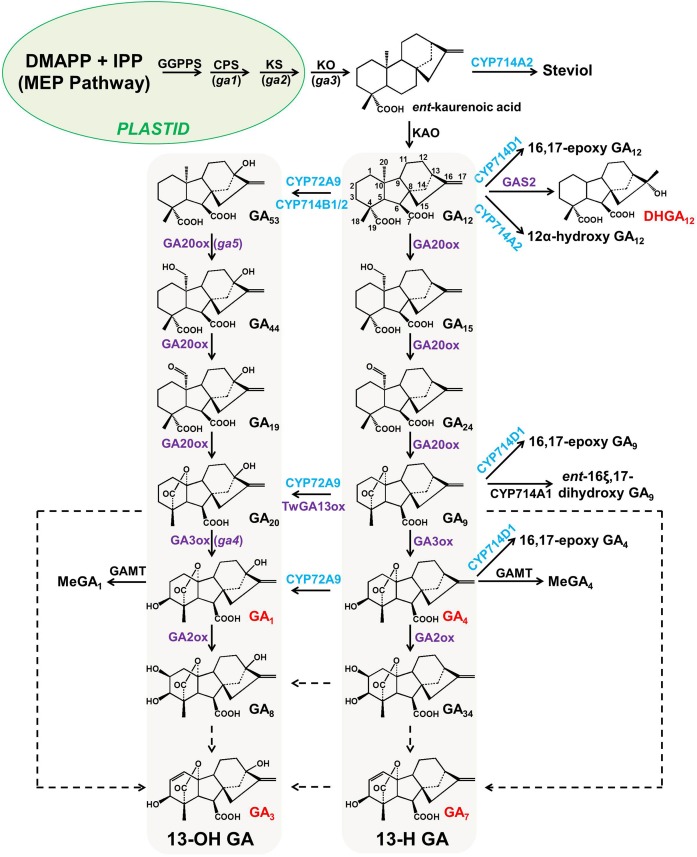
Simplified GA metabolic pathway in flowering plants. All enzymes mapped to the GA metabolic pathway, except GA13ox from *Tripterygium wilfordii*, were verified with enzymatic assays and the chemical profiling of transgenic plants. P450s (ER membrane-associated proteins) in this pathway are highlighted in blue, while 2-oxoglutarate-dependent dioxygenases (2-ODDs, cytosolic proteins) are highlighted in purple. All dashed lines indicate enzymatic a step that has not yet been characterized at the genetic level in any flowering plant. The genes/enzymes characterized from Arabidopsis and rice are marked in black and blue, respectively. The carbon backbone of GA_12_ is labeled with numbers, and the bioactive GAs (GA_1_, GA_3_, GA_4_, GA_7_, and DHGA_12_) are marked in red. The Arabidopsis GA-deficient mutants (*ga1* to *ga5*) used for map-based cloning are shown in parentheses next to the corresponding genes. CPS, *ent*-copalyl diphosphate synthase; GAMT, GA methyltransferase; GAS2, gain of function in ABA-modulated seed germination 2; GGPPS, geranylgeranyl diphosphate synthase; KAO, *ent*-kaurenoic acid oxidase; KO, *ent*-kaurene oxidase; KS, *ent*-kaurenoic acid synthase; MEP, 2-*C*-methyl-*D*-erythritol 4-phosphate.

Gibberellins (GAs), a type of 6-5-6-5 tetracyclic diterpenoid, are essential phytohormones for plant growth and development. The more than 130 GAs discovered to date have been classified into two groups based on their number of carbon atoms: C_19_-GAs (with one carboxylic group at the C-7 position, e.g., GA_20_ and GA_9_ in Figure 1) and C_20_-GAs (with two carboxylic groups at the C-7 and C-19 positions, respectively, e.g., GA_12_ and GA_53_ in Figure 1). GAs have also been classified into two groups based on other chemical structural criteria: 13-H GAs (hydrogen at the C-13 position) and 13-OH GAs (hydroxyl group at the C-13 position). Plant biologists consider GA_4_, GA_1_ (also known as 13-OH GA_4_), GA_7_, and GA_3_ (also known as 13-OH GA_7_) to be the only common bioactive GAs in flowering plants. The bioactivity of GA_1_ in plants is ∼1000-fold lower than that of GA_4_ (Eriksson et al., 2006).

## Ga Biosynthesis in Flowering Plants

The GA metabolic pathway at the molecular level has been extensively investigated using both forward and reverse genetics strategies. Both strategies used on GA metabolism started with the dwarfism phenotype caused by bioactive GA deficiency (e.g., so-called GA-sensitive mutants). A forward genetics strategy, in which a mutant population is scored by the dwarf phenotype change followed by map-based cloning of the causal genes, has been applied to GA biosynthesis gene identification in Arabidopsis, from GGPP to GA_4_, for a long time. Thus far, five GA-sensitive Arabidopsis mutants (*ga1* to *ga5*) have been used to determine the causal genes: *GA1* encodes CPS (*ent*-copalyl diphosphate synthase; EC 5.5.1.13), *GA2* encodes KS (*ent*-kaurene synthase; EC 4.2.3.19), *GA3* encodes KO (*ent*-kaurene oxidase; EC 1.14.13.79), and *GA4* and *GA5* encode GA 3β-hydroxylase and GA 20-oxidase respectively (Yamaguchi, 2008; Hedden and Sponsel, 2015). As there are 10 *GGPPS* (geranylgeranyl diphosphate synthase; EC 2.5.1.29) or *GGPPS*-like genes in the Arabidopsis genome, it is not clear which *GGPPS* gene(s) is responsible for GA biosynthesis. A recent study demonstrated that GGPPSL1, 2, 3, 4, and 11 are *bona fide* GGPPS enzymes in Arabidopsis (Nagel et al., 2015; Wang et al., 2016). Combined with the subcellular localization (GGPPSL2 and 11 are plastidial proteins) and tissue specificity (*GGPPSL11* showed a much higher level than *GGPPSL2* in all tested Arabidopsis tissues) information, it can be inferred that *GGPPSL11* (*At4g36810*) is likely the key player in providing GGPP for GA biosynthesis, although *GGPPSL11* also functioned as a GGPP provider for chlorophyll and carotenoid biosynthesis in Arabidopsis (Beck et al., 2013; Vranova et al., 2013; Chen et al., 2015; Wang et al., 2016). The knock-down mutant of *GGPPSL11* (SALK_140601, T-NDA inserted at the promoter region) showed a dwarf phenotype and pale-green leaves, while knock-out mutants of *GGPPSL11* showed a seedling-lethal phenotype (Chen et al., 2015). Notably, endogenous GAs were not measured in any tissue of either *GGPPSL2* or *GGPPSL11* mutants.

Thanks to the work of phytochemists, 136 naturally occurring GAs have been identified thus far (Hedden, 2019). However, no novel GAs have been reported in plants for a long time. Recently, Liu et al. (2019) reported a novel gibberellin, DHGA_12_ (chemical structure shown in Figure 1), which possesses a C_20_ skeleton structure and can promote seed germination and seedling establishment in Arabidopsis. DHGA_12_, like other bioactive GAs (e.g., GA_1_, GA_3_, GA_4_, and GA_7_), has the ability to bind the GA receptor (GID1), although it has a lower affinity to GID1 than GA_4_. GAS2, a 2-oxoglutarate-dependent dioxygenase (2-ODD), was also identified to catalyze GA_12_ into DHGA_12_ (Liu et al., 2019). However, the ABA (abscisic acid) level was significantly reduced in *GAS2* overexpression lines, which complicates the explanation of how *GAS2* promotes seed germination. Does the increase in DHGA_12_ (twofold) compensate for the decrease of GA_4_ (10-fold) in the *GAS2* overexpression lines? Furthermore, DHGA_12_ or GA_4_ could only partially rescue the phenotype of cotyledon greening and hypocotyl elongation in *gas2* mutants, indicating that there were other factors involved in these processes. Given the promiscuity of plant 2-ODDs (Mitchell and Weng, 2019), it seems that the activity of GAS2 is not restricted to GA_12_ oxidation to form DHGA_12_ but that it acts on other unknown substrates. The possibility of whether GAS2 is also involved in ABA inactivation via oxidation remains to be clarified. Comprehensive metabolite analysis of *GAS2* transgenic lines will provide valuable evidence to elucidate the *GAS2* functions in Arabidopsis.

As shown in Figure 1, it remains unknown how the other two bioactive GAs, GA_3_, and GA_7_, are biosynthesized in plants. GA_4_-desaturase (GA_4_-DES), which also belongs to the 2-ODD family and which converts GA_4_ to GA_7_ (Figure 1), has been functionally identified from *Gibberella fujikuroi* (Tudzynski et al., 2003; Bhattacharya et al., 2012). The overexpression of *GA_4_-DES* in plants resulted in a 20-fold higher concentration of GA_3_ along with an ∼70-fold reduction in GA_4_. Moreover, the resulting transgenic plants showed a substantial growth increase, which reflected the increase in the total bioactivity of GA *in planta* (Bhattacharya et al., 2012). However, the functional homologs of fungal *GA_4_-DES* have not been characterized from plants to date. The Peter Hedden group recently demonstrated that two GA 3-oxidases from southern wild cucumber (*Marah macrocarpus*), MmGA3ox1 and MmGA3ox2, were involved in GA_3_/GA_7_ production (from GA_9_ and/or GA_20_) (Ward et al., 2010). Notably, these results do not exclude the possibility that plants evolved other enzyme systems (a case of convergent evolution) to produce GA_3_/GA_7_, which merits further investigation.

## Ga Catabolism in Flowering Plants

**FIGURE 2:**
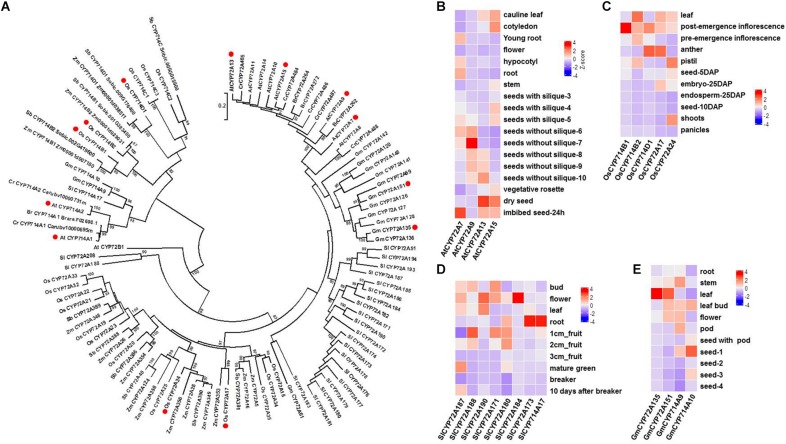
Characterization of GA oxidases encoded by *P450* genes in flowering plants. **(A)**. Phylogenetic analysis of plant GA oxidases encoded by P450 genes using MEGA 6.0 (Tamura et al., 2013). A total of 102 P450 proteins (members of the CYP72A and CYP714 subfamilies) were obtained from 8 representative species in plant evolutionary history. P450 proteins with GA oxidase activity are marked with red circles. Bootstrap values (based on 500 replicates) >80% are shown for corresponding nodes. At, *Arabidopsis thaliana*; Br, *Brassica rapa*; Cr, *Capsella rubella*; Gm, *Glycine max*; Os, *Oryza sativa*; Sb, *Sorghum bicolor*; Sl, *Solanum lycopersicum*; Zm, *Zea mays*. **(B)** Tissue specificity of four *CYP72A* genes in Arabidopsis. Data were extracted from the gene expression map of Arabidopsis development (http://arabidopsis.org). **(C)** Tissue specificity of three *CYP714* genes and two *CYP72A* genes in rice. RNA-Seq (FPKM) data were extracted from the Rice Genome Annotation Project (http://rice.plantbiology.msu.edu). **(D)** Tissue specificity of seven *CYP72A* genes and one *CYP714A* gene in tomato plants. Data were extracted from the Tomato Genome Consortium (Sato et al., 2012). **(E)** Tissue specificity of two *CYP72A* genes and two *CYP714A* genes in soybean plants. Data were extracted from Shen et al. (Shen et al., 2014).

Previous studies have shown that not only GA biosynthesis but also GA catabolism (deactivation) play important roles in GA bioactivity homeostasis *in planta*. Thus far, three types of GA deactivation enzymes and their encoding genes have been identified, including GA 2-oxidase (belonging to the 2-ODD family; seven *GA2ox* genes in Arabidopsis and ten *GA2ox* genes in rice) (Rieu et al., 2008), GA methyltransferase (*GAMT1/2* from Arabidopsis) (Varbanova et al., 2007), and GA 16,17-oxidase (*CYP714D1/EUI* from rice) (Zhu et al., 2006). Phylogenetic analysis shows that there are three CYP714 subclades (CYP714B, C, and D) in the rice genome (Figure 2A). It is plausible to speculate that other members of the CYP714 family might be involved in GA deactivation in plants. Recently, reverse genetic studies in rice have suggested a dominant role for CYP714B1 and CYP714B2 in reducing GA activity through 13-hydroxylation of GA_12_ to form GA_53_ (Magome et al., 2013). Interestingly, both *cyp714d1* mutant and *cyp714b1/cyp714b2* double mutant rice display an elongated uppermost internode, where these three *P450*s are highly expressed, at the heading stage. The measurement of endogenous GAs showed that the levels of 13-OH GAs were decreased, while the levels of the 13-H GAs were increased in the *cyp714b1/cyp714b2* double mutant. However, there are no members of the CYP714D and CYP714B subfamily in Arabidopsis. The closest analogs to CYP714D1 and CYP714B1/B2 in Arabidopsis are CYP714A1 and CYP714A2 (Figure 2A). Both are GA deactivation enzymes, at least *in vitro*: CYP714A1 catalyzes the conversion of GA_12_ into 16-carboxylated GA_12_, while CYP714A2 catalyzes the conversion of *ent*-kaurenoic acid into steviol (*ent*-13-hydroxy kaurenoic acid) (Zhang et al., 2011; Nomura et al., 2013). In contrast to their rice counterparts, knockdown of both *CYP714A1* and *CYP714A2* led to increased bioactive GA_4_ and no change in GA_1_ when compared to those in wild-type plants. Together with the wide occurrence of GA_1_ in flowering plants, these results indicate that *CYP714A1* and *CYP714A2* are not the main contributors to GA 13-hydroxylation and that other genes are responsible for 13-OH GA production in Arabidopsis.

More recently, CYP72A9 (encoded by *At3g14630*) was reported to catalyze the 13-H GAs (GA_12_, GA_9_, GA_4_) into 13-OH GAs (GA_53_, GA_20_, GA_1_), which is inconsistent with the traditional view that GA_4_ and GA_1_ are biosynthesized in parallel by GA20ox and GA3ox in flowering plants, from GA_12_ and GA_53_, respectively (Figure 1; He et al., 2019). It was unexpected to discover that *CYP72A9* encoded GA 13-hydroxylase, as *CYP72A9* was located in one geranylfarnesyl pyrophosphate synthase (C25)-sesterterpene synthase-P450 (*GFPPS-sesterTPS-P450*) gene cluster. Eight tandem duplicated CYP72As (CYP72A7, At3g14610; A8, At3g14620; A9, At3g14630; A10, At3g14640; A11, At3g14650; A13, At3g14660; A14, At3g14680; A15, At3g14690) are located together at the same chromosome region. These eight *CYP72A*s were first thought to be involved in sesterterpene (C25) biosynthesis (Wang et al., 2016; Shao et al., 2017; Chen Q. et al., 2019). Plants overexpressing *CYP72A9* exhibited semi-dwarfism, which was caused by a significant reduction in GA_4_ levels. Moreover, *CYP72A9* was expressed predominantly in developing seeds in *Arabidopsis*, and *cyp72a9* mutants showed a deficiency of GA_1_ but an increase of GA_4_ in the siliques. *CYP72A9* was further proven to be involved in primary seed dormancy (He et al., 2019). The other three Arabidopsis CYP72A members (A7, A13, and A15) also showed GA 13-hydroxylase activity, but overexpression plants did not show a semi-dwarf phenotype, which merits further investigation of the physiological functions of these genes *in planta*. Homologs of CYP72A9 in other *Brassicaceae* plants, such as CYP72A262 in *Brassica rapa* and CYP72A484 in *Capsella rubella*, also have GA 13-hydroxylase activity, although with slightly different substrate preferences (He et al., 2019). It appears that the recruitment of GA deactivation enzymes shows different tissue specificity in different plant species, at least at the family level: *CYP714D1* and *CYP714B1/B2* are highly expressed in the uppermost internode of rice (*Poaceae* family), while all GA deactivation genes (*CYP72A9, GAMT1/2*, and *CYP714A1*/*A2*) are predominantly expressed in the developing siliques/seeds of Arabidopsis (*Brassicaceae* family). As both CYP714 and CYP72A are widely distributed in flowering plants, it will be interesting to determine the role of each member of both P450 subfamilies in contributing to GA homeostasis in different plant species (Figure 2). The tissue specificity of potential GA genes from the *CYP714* and *CYP72A* subfamilies also suggested the functional divergence of GA deactivation in different plant species (the genes from Arabidopsis, rice, soybean, and tomato are shown in Figures 2B–E). For example, CYP72A135 has been demonstrated to catalyze the conversion of GA_9_ to GA_20_ (He et al., 2019), and *CYP72A135* was predominantly expressed in leaves in soybean plants (Figure 2D). Thus, further investigation is needed to test whether *CYP72A135* is involved in leaf development in soybean.

Zhang et al. recently reported another type of GA13-hydroxylase, TwGA13ox, from *Tripterygium wilfordii* (*Celastraceae* family). TwGA13ox, in contrast to the P450s characterized in rice and Arabidopsis, is a 2-ODD enzyme, like GA20ox, GA3ox, and GA2ox (Figure 1; Zhang et al., 2019). *TwGA13ox* was highly expressed in root phloem and could be induced by methyl jasmonate (MeJA) treatment. TwGA13ox specifically catalyzes the conversion of GA_9_ to GA_20_ *in vitro* but does not catalyze the formation of GA_1_ from GA_4_. It is noteworthy that the more detailed biochemical and physiological function of *Tw*GA13ox has not yet been determined in *Tripterygium wilfordii*. Additionally, GA13-hydroxylase activity of the 2-ODD enzyme has previously been reported in cucumber plants (*Cucumis sativus* L.) (Lange et al., 2013). However, it is unlikely that 2-ODD enzymes are the main contributor to 13-OH GA production in flowering plants, as the above-mentioned P450s are responsible for 13-OH GA production in rice and Arabidopsis, which has been shown at the biochemical and genetic levels.

## Qualitative and Quantitative Analysis of Endogenous GAs

Due to the very low level of endogenous GAs, accurate quantitative measurement plays an important role in elucidating the GA metabolic pathway in plants. Currently, triple quadrupole MS (mass spectrometry) with MRM (multi-reaction monitoring) is the most frequently used operation mode for the quantitative analysis of target GAs (Pan et al., 2010; Urbanova et al., 2013; Chu et al., 2017). In most cases, the GAs shown in Figure 1 can be quantitatively measured using the LC-QQQ-MS/MS technique combined with GA enrichment using SPE (solid-phase extraction) sorbents. For the qualitative discovery and identification of unknown GAs, MS, and MS/MS full scans are commonly used to obtain more structural information. Sometimes, high-resolution MS, such as Q-TOF (quadrupole time-of-flight), Orbitrap, or FT-ICR (Fourier transform ion cyclotron resonance), is necessary to derive the molecular formulae of the precursor and product ions. The purification of a novel GA from plants and the elucidation of its absolute chemical structure is always a time-consuming and laborious procedure.

## Synthetic Biology for Bioactive GA Adjustment in Plants

Since GAs play important roles in regulating plant growth and development, the GA genes described here represent a promising toolkit with which synthetic biologists can adjust bioactive GA levels to manipulate plant traits. The most famous GA gene used for crop improvement is the “Green Revolution” gene in rice: the “dwarfing” rice caused by the knock-out of *Os20ox2* (semi-dwarfing allele, *sd-1* mutant) showed high resistance to lodging stress and significantly increased the yield (to support the heavy grain of the high-yielding varieties), in combination with the application of large amounts of fertilizer and pesticides (Sasaki et al., 2002; Spielmeyer et al., 2002; Hedden, 2003). In Arabidopsis, a previous study showed that overexpression of *AtCPS* (*At4g02780*) in Arabidopsis leads to an increase in *ent*-kaurene/*ent*-kaurenoic acid and GA_12_ but does not affect the level of bioactive GAs or cause a phenotype change (Fleet et al., 2003). Moreover, the overexpression of *AtGA20ox* (*At4g02780*) in Arabidopsis (Col-0) leads to an increase in bioactive GAs and the GA-overproduction phenotype (Huang et al., 1998). Notably, there were higher concentrations of GA_1_ and GA_20_ in *AtGA20ox* OE plants than in wild-type seedlings, whereas the concentration of GA_4_ remained approximately the same. The constitutive promoters, coupled with additional copies of the GA genes, were applied in all of the above-mentioned cases in Arabidopsis. All these results also confirmed that GA genes are sensitive to endogenous bioactive GA changes: GA biosynthesis genes are usually upregulated when GA_4_ decreases, while GA catabolism genes are downregulated under the same conditions, and *vice versa* (He et al., 2019). Synthetic biology could thus be used to replace native promoters with new tunable promoters that active/deactivate in response to endogenous bioactive GA and even other internal/external signals (Kassaw et al., 2018). At the enzyme level, the targeted near-saturated mutagenesis of GA genes, especially those downstream of GA_12_ formation (multiple copies with different tissue-specificity), using CRISPR-based editing could be applied to fine-tune bioactive GA levels to achieve ideal plant traits (Chen K. L. et al., 2019; Eshed and Lippman, 2019). After quantitatively characterizing these GA components *in planta*, other GA-related factors, such as signal transduction and interactions with other phytohormones, etc., will be considered and re-designed to drive GA synthetic biology forward.

## Author Contributions

GW and JH conceptualized the content of the manuscript. JH wrote the first draft of the manuscript. GW finalized the manuscript with contributions from JH, PX, XM, and JC.

## Conflict of Interest

The authors declare that the research was conducted in the absence of any commercial or financial relationships that could be construed as a potential conflict of interest.
